# Antidepressant-like effects of a chlorogenic acid- and cynarine-enriched fraction from *Dittrichia viscosa* root extract

**DOI:** 10.1038/s41598-022-04840-9

**Published:** 2022-03-07

**Authors:** Kateryna Murlanova, Netanela Cohen, Anna Pinkus, Liudmila Vinnikova, Mikhail Pletnikov, Michael Kirby, Jonathan Gorelick, Elyashiv Drori, Albert Pinhasov

**Affiliations:** 1 Department of Molecular Biology, Ariel University, 4070000 Ariel, Israel; 2 Department of Psychiatry and Behavioral Sciences and Solomon H. Snyder Department of Neuroscience, Johns Hopkins University School of Medicine, Baltimore, MD 21205 USA; 3 Department of Physiology and Biophysics, Jacobs School of Medicine and Biomedical Sciences, State University of New York at Buffalo, Buffalo, NY 14203 USA; 4 Department of Chemical Engineering, Ariel University, 4070000 Ariel, Israel; 5 Eastern Regional R&D Center, Ariel University, 4070000 Ariel, Israel; 6 Adelson School of Medicine, Ariel University, 4070000 Ariel, Israel

**Keywords:** Biochemistry, Drug discovery, Neuroscience, Psychology

## Abstract

*Dittrichia viscosa* is a perennial Mediterranean plant used in traditional medicine for “calming purposes”, pointing at a possible antidepressant activity of the plant. We conducted chromatographic and bioassay-guided fractionation of *D. viscosa* root extract to isolate a specific fraction (fraction “K”) with antidepressant-like characteristics in vivo and strong antioxidant properties in vitro. A single dose of “K” reduced immobility time in the forced swim test with a mouse model possessing a depressive-like phenotype. Neurochemical profiling for 5-hydroxytryptamine (5-HT) and its primary metabolite, 5-hydroxyindoleacetic acid (5-HIAA), in prefrontal cortex and hippocampus of “K”-treated mice showed reduction in 5-HIAA, indicative of either serotonin uptake transporter or monoamine oxidase-A inhibition, as well as slight increases in 5-HT content. These neurochemical alterations, as well as the behavioral changes observed, were comparable to the effects of paroxetine. “K” also protected PC12 cells in a H_2_O_2_ cytotoxicity assay, thus demonstrating antioxidant properties, yet paroxetine augmented oxidative damage and cell death. Identification of the main compounds in “K” by high-performance liquid chromatography-tandem mass spectrometry (HPLC–MS/MS) indicated that chlorogenic acid and cynarine comprised 87% of the total components. *D. viscosa* root extract appears to produce antidepressant and cytoprotective effects and may serve as an attractive alternative to standard therapies for depression.

## Introduction

The global prevalence of depressive disorders is rapidly growing^1^ and is a leading cause of disability^2^. However, treatment strategies targeted to depression have a high failure rate and remain limited by their side effects, slow response, and low efficacy^3,4^. Thus, there is a real need for the development of novel, safe agents with high pharmacological efficacy.

It is estimated that around eighty percent of the global population relies on herbal medicinal products as a primary source of healthcare^5^. A surge in acceptance and public interest in natural therapies both in developing and developed countries contributes to the development of novel pharmacologically active compounds derived directly or indirectly from plants. In the search for new drug entities, remarkable efforts are spent on chromatographic separation and mass spectrometry techniques allowing single-plant secondary metabolites to be efficiently isolated from their native hosts and their chemical structures to be unequivocally determined. Subsequent bioassay-guided fractionation of plant extracts and high-throughput activity screening using natural product libraries may result in the discovery of a variety of therapeutic agents, many of which are widely used in the clinic today^6^. Standardization of extracts, their fractions, or single active molecules is another vital issue ensuring qualitative and quantitative values, efficacy, and reproducibility of the pharmacological effect^7^.

Numerous plant natural products have successfully entered the clinic as complementary and alternative medicines for the treatment of psychiatric disorders, for example, crude and standardized extracts of *Hypericum perforatum* L.^8^, *Valeriana officinalis* L.^9^, *Melissa officinalis* L., and *Verbena officinalis* L.^10^. Notably, many plant-originated drugs were discovered through their use in traditional medicine. Ethnobotanical research, followed by the use of advanced technological tools, molecular and cellular methodology, and valid animal models, serves as a platform for the development of novel active compounds^11,12^.

*Dittrichia viscosa* (L.) Greuter (Asteraceae), commonly known as False Yellowhead or Woody Fleabane, is a herbaceous perennial Mediterranean plant species^13^ formerly belonging to the genus *Inula*^14^. According to ethnopharmacological surveys and reviews of medicinal herbs in Israel, *D. viscosa* is commonly used in Arabic traditional medicine^15–18^. The traditional uses of *D. viscosa* [Arabic name, tayun (sticky) davik] are vast including treatment of skin diseases, rheumatic pains, prevention of infections, reduction of high blood pressure, reduction of high sugar levels, infertility, and other uses^18,19^. Among different traditional applications of *D. viscosa,* it is popularly used for “calming purposes”, muscle relaxation, analgesia, and fatigue treatment^15,17,18,20^. Fatigue is a residual symptom of depression^21,22^ and many commercially-available antidepressants possess relaxing and analgesic properties^23,24^, suggesting a possible antidepressant-like potential in *D. viscosa*.

In this study, we aimed to determine whether an extract of *D. viscosa* root does indeed have the properties ascribed to it in the ethnobotanical literature, namely antidepressant effects. Further, we aimed to perform a chromatographic separation and bioassay-guided fractionation of the extract, selecting for fractions with increased pharmacological properties, as an initial effort to identify plant compounds of pharmaceutical potential matching the reported effects. We identified one fraction (named fraction “K”) which exhibited cytoprotective qualities as well as antidepressant-like effects in a mouse model with depressive-like phenotype developed in our laboratory^25,26^. Fraction “K” was evaluated by mass spectral analysis for bioactive compounds and two prominent chemicals were identified: chlorogenic acid and cynarine. Here, we present evidence for the pharmacological potential of *D. viscosa* root extract as an antidepressant natural product which contains at least two compounds of known pharmacology. Ongoing work in our lab is focused on identifying other molecules of therapeutic potential in fraction “K” of *D. viscosa* root.

## Results

### Crude extract did not exert a toxic effect in PC12 cells but failed to protect against oxidative stress

**Figure 1:**
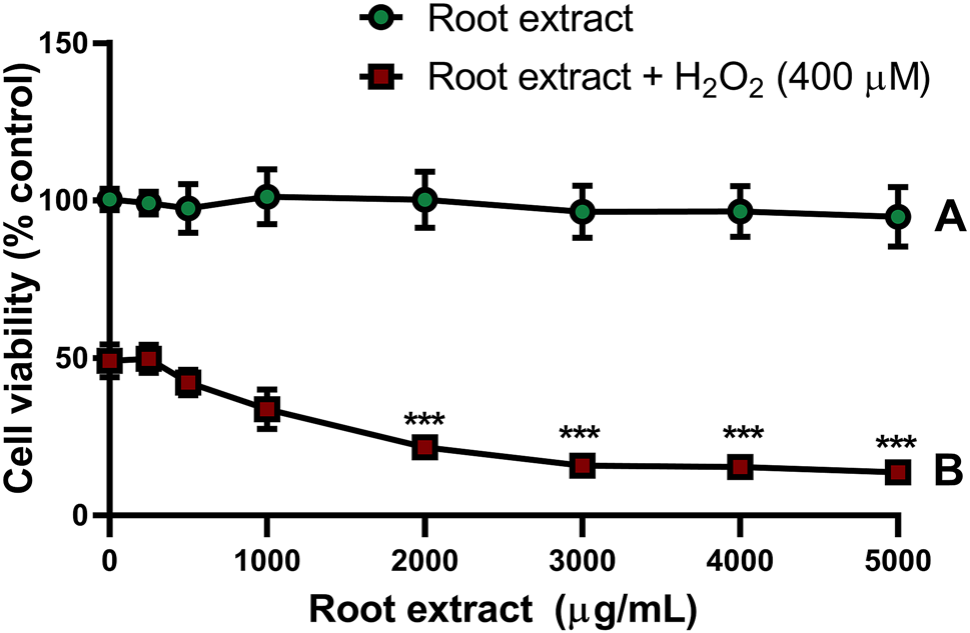
Effects of crude *D. viscosa* root extract on PC12 cell viability. Mean cell viability (± SD) measured by XTT assay for (**A**) PC12 cells incubated with concentrations of crude *D. viscosa* root extract (0–5000 μg/mL) for 14 h or (**B**) PC12 cells pretreated with concentrations of crude *D. viscosa* root extract (0–5000 μg/mL) for 2 h followed by incubation for 12 h with H_2_O_2_ (400 μM). Two-way ANOVA: crude root extract concentration, *p* < 0.0001; pretreatment, *p* < 0.0001; interaction, *p* < 0.0001. Dunnett’s test: ***, *p* < 0.0001.

PC12 cells incubated with a broad range (250–5000 μg/mL) of crude root extract concentrations did not present with cytotoxic or proliferative effects (Dunnett’s test: *p* > 0.05; Fig. 1). However, pretreatment with the crude root extract failed to protect PC12 cells against 400 μM H_2_O_2_-induced cytotoxicity and concentration-dependently reduced cell viability by 72.6% (Dunnett’s test [1000–5000 μg/mL]: *p* < 0.0001). Two-way ANOVA: Crude root extract concentration: F[7,144] = 38.4, *p* < 0.0001; H_2_O_2_ treatment: F[1,144] = 4962, *p* < 0.0001; interaction: F[7,144] = 26.99, *p* < 0.0001). Effects of crude root extract with or without the presence of H_2_O_2_ differed (Sidak test [A vs. B by concentration], *p* < 0.0001).

### HPLC-guided fractionation resulted in 14 fractions exhibiting diverse properties in a H_2_O_2_-induced cytotoxicity model

As shown (Fig. 1B) root extract showed no protective effect against H_2_O_2_ cytotoxicity, however we assumed (taking in mind the behavioral effect in FST, Fig. 4) that extract fractionation may result in identification of active compounds with more potent beneficial pharmacological properties. HPLC-guided separation of crude extract (Suppl. Fig. 1A) resulted in 14 fractions. Each fraction contained between 2 to 8 peaks (Suppl. Fig. 1B, representative fraction), with each peak corresponding to at least one compound.

**Figure 2:**
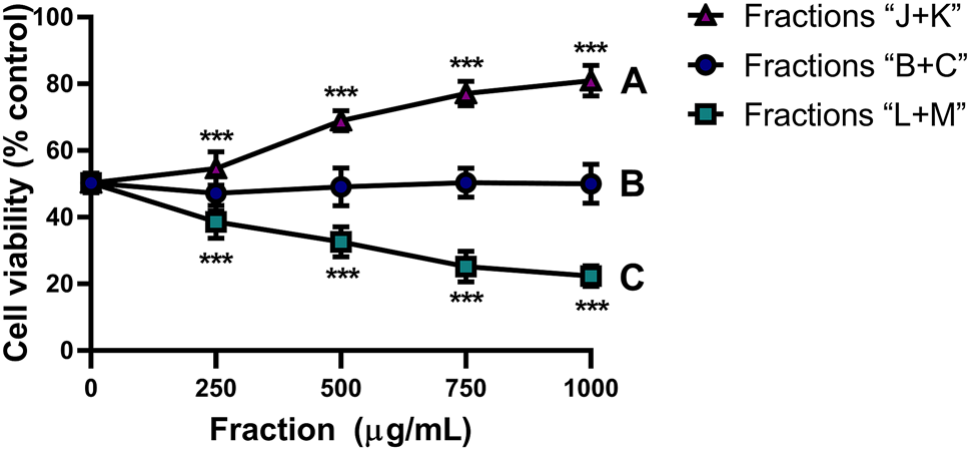
Effect of root extract fraction combinations on PC12 cell viability. Mean cell viability (± SD) measured by XTT assay of PC12 cells pretreated for 2 h with concentrations of combined root extract fractions (0–1000 μg/mL) (**A**) “J + K”, (**B**) “B + C”, or (**C**) “L + M” followed by 12 h of oxidative stress (400 μM H_2_O_2_). Two-way ANOVA: fraction, *p* < 0.0001; concentration, *p* < 0.0001; interaction, *p* < 0.0001. Dunnett’s test: *p* = 0.0001, ***.

To identify fractions with beneficial biological activity, we assumed that cytoprotective effects would accompany the FST behavioral effects observed with the crude root extract. Combinations of separated fractions were tested in a concentration range similar to those of the crude extract (0–1000 μg/mL) in the XTT cell viability oxidative stress assay (400 μM H_2_O_2_) with PC12 cells (Fig. 2). Our initial assumptions appeared to be correct. Cell viability effects of all fraction combination treatments differed substantially (Two-way ANOVA followed by Tukey’s HSD: Fraction: F[2,135] = 703.5, *p* < 0.0001; concentration: F[4,135] = 4.897, *p* = 0.0010; interaction: F[8,135] = 79.81, *p* < 0.0001; Tukey’s HSD: 250–1000 μg/mL, all fractions, *p* < 0.0001, ***).

Combination of fractions “B + C” and “L + M” were not protective against oxidative stress, with fraction “B + C” conferring no cytoprotective effect and fraction “L + M” concentration-dependently reducing cell viability (Dunnett’s test: *p* = 0.0001, all concentrations). Fraction “J + K” concentration-dependently increased cell viability by 31% over basal H_2_O_2_-induced oxidative stress (Dunnett’s test: *p* = 0.0001, 500–1000 μg/mL). Thus, fractions “J” and “K” were further separated and evaluated separately against H_2_O_2_-evoked cell death.

### Fraction “K” markedly protected cells against H_2_O_2_-induced cytotoxicity

**Figure 3:**
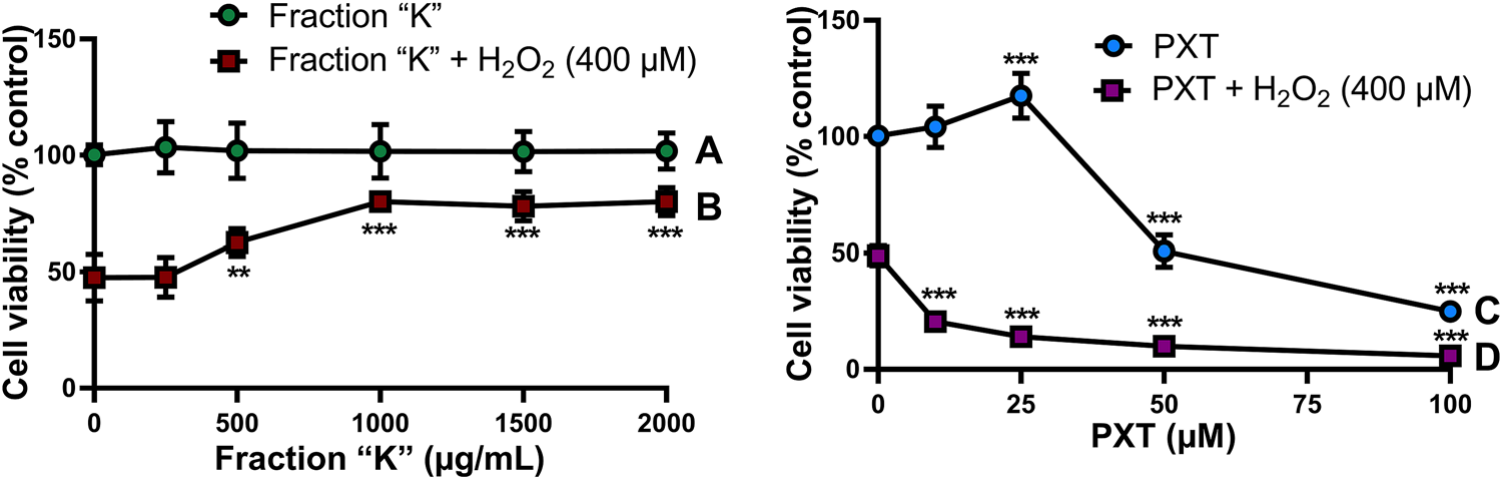
Comparative effects of fraction “K” and PXT on cell viability. Mean cell viability (± SD) measured by XTT assay for (**A**) PC12 cells incubated with concentrations of fraction “K” (0–2000 μg/mL) for 14 h or (**B**) PC12 cells pretreated with concentrations of fraction “K” (0–2000 μg/mL) for 2 h followed by incubation for 12 h with H_2_O_2_ (400 μM). Mean cell viability (± SD) measured by XTT assay for (**C**) PC12 cells incubated with concentrations of PXT (0–100 μM) for 14 h or (**D**) PC12 cells pretreated with concentrations PXT (0–100 μM) for 2 h followed by incubation for 12 h with H_2_O_2_ (400 μM). Two-way ANOVA: (**A,B) **Concentration, *p* < 0.0001; pretreatment, *p* < 0.0001; interaction, *p* < 0.0001. Sidak: all treatments, *p* < 0.0001; Dunnett’s test: (**B)** 500 μg/mL, *p* = 0.0006; 1000–2000 μg/mL, *p* < 0.0001. (**C,D)** Concentration, *p* < 0.0001; pretreatment, *p* < 0.0001; interaction, *p* < 0.0001. Sidak: all treatments, *p* < 0.0001; Dunnett’s test: (**C)** 25–100 μM, *p* < 0.0001; (**D)** all treatments, *p* < 0.0001. **, *p* = 0.0006; ***, *p* < 0.0001.

In the XTT cell viability assay, responses between PC12 cells treated with different concentrations of fraction “K” with and without H_2_O_2_ differed (Two-way ANOVA with Sidak test: Concentration, F[5,108] = 17.44, *p* < 0.0001; pretreatment, F[1,108] = 548.2, *p* < 0.0001; interaction, F[5,108] = 17.76, *p* < 0.0001; Sidak, all treatments, *p* < 0.0001; Fig. 3). Fraction “K” did not cause a proliferative effect in PC12 cells, but dose-dependently increased cell survival under H_2_O_2_-induced oxidative stress to a maximum of 68.3% at 2000 μg/mL (Dunnett’s test: 500 μg/mL, *p* = 0.0006; 1000–2000 μg/mL, *p* < 0.0001). In contrast, fraction “J” produced a deleterious effect on cell viability (Two-way ANOVA: Concentration, F[4,50] = 3.015, *p* = 0.0264; H_2_O_2_ treatment, F[1,50] = 1857, *p* < 0.0001; interaction, F[4,40] = 5.088, *p* = 0.0016; Sidak test: *p* < 0.0001, all treatments; Dunnett’s test: Fraction “J”, *p* > 0.05; fraction “J” + H_2_O_2_, 250 μM, *p* = 0.0005; 500 μM, *p* < 0.0001; 1000–2000 μM, *p* < 0.016; Suppl. Fig. 2). Treatment of PC12 cells with paroxetine (PXT), produced cytoprotective effects at lower concentrations (≤ 25 μM), however increasing concentrations were cytotoxic, reducing cell viability by 88.2% at a 100 μM concentration (Dunnett’s test: 25–100 μM, *p* < 0.0001). Interestingly, all tested concentrations of PXT, including non-cytotoxic ones, markedly reduced cell survival in the H_2_O_2_-induced cytotoxicity model (Dunnett’s test: 1000–2000 μM, *p* < 0.0001).

### Acute and sub-chronic D. viscosa root extract administration decreased mouse immobility time in the forced swim test (FST)

**Figure 4:**
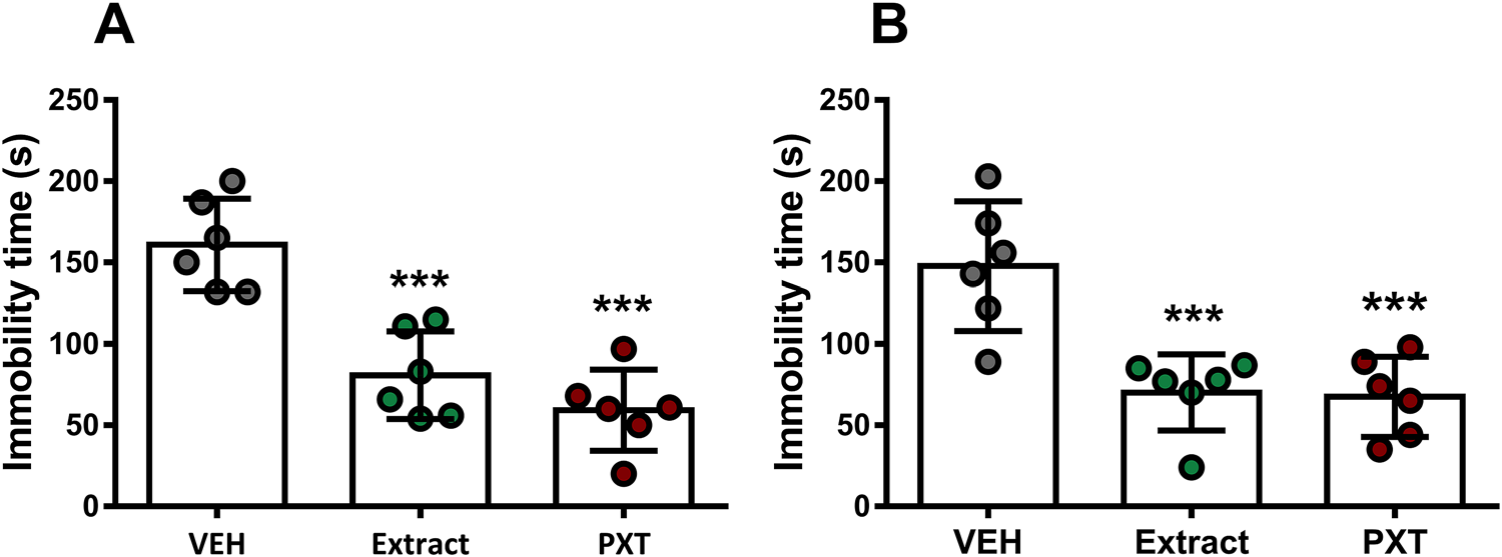
Effect of crude *D. viscosa* root extract and PXT on immobility time in FST. (**A**) Single or (**B**) sub-chronic (14-day, daily) administration of crude *D. viscosa* root extract (5 mg/kg i.p.) or PXT (10 mg/kg i.p.) produced reductions in immobility time in FST (One-way ANOVA with Dunnett’s test: A: *p* < 0.0001; B: *p* < 0.0001). Data are expressed as mean (± SD). Abbreviations are as follows: VEH, vehicle; Extract, crude *D. viscosa* root extract; PXT, paroxetine. ***, *p* < 0.0001.

The behavioral effect of *D. viscosa* extracts was examined in submissive (Sub) mice, previously validated as a mouse model of depressive-like behavior^26–28^. Initial screening of *D. viscosa* root extract revealed that single and sub-chronic (14 days, daily) 5 mg/kg i.*p*. injection of root crude extract caused reduction in immobility time in the FST (Single dose: one-way ANOVA: F[2,25] = 23.95, *p* < 0.0001; sub-chronic dosing: F[2,15] = 13.64, *p* = 0.0004; Fig. 4). These results were similar to the effect of PXT (10 mg/kg), a widely prescribed selective serotonin reuptake inhibitor (SSRI) that was used as a positive control (Fig. 4). Neither the herbal extract nor PXT produced tachyphylaxis, indicated by similar behavioral responses regardless of single or 14-day daily treatments.

### Fraction “K” produced antidepressant-like effects similar to paroxetine

**Figure 5:**
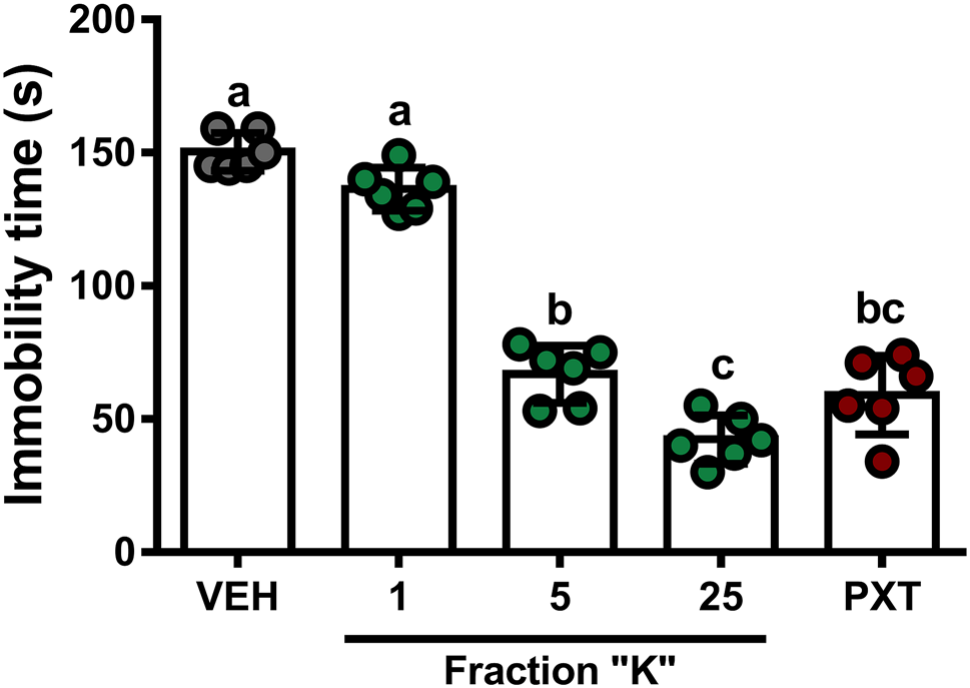
Effect of fraction “K” and PXT on immobility time of mice in the FST. Mean immobility time (± SD) in FST following a single i.p. dose of fraction “K” (0–25 mg/kg) or PXT (10 mg/kg) differed from both 1.0 mg/kg fraction “K” treatment and control (One-way ANOVA, *p* < 0.0001). Different letters indicate significant differences (Tukey’s HSD, *p* < 0.0001 all treatments except 25 mg/kg fraction “K” vs. PXT [*p* = 0.0021]).

Administration of single doses of fraction “K” to Sub mice subjected to the FST indicated similar reductions of depressive-like behavior comparable to the comparison dose of PXT (10 mg/kg) (One-way ANOVA, F[4,45] = 135.5, *p* < 0.0001; Fig. 5). Doses of 5 mg/kg or 25 mg/kg fraction “K” reduced mouse immobility times by 55.5% and 71.8%, respectively, which were comparable with the 60.7% reduction in immobility times using PXT. Fraction “K” doses as low as 1.0 mg/kg did not produce any measurable antidepressant-like effect in the FST. In experiments with these doses of fraction “K” in open field assessment, no alterations of locomotory activity were observed (One-way ANOVA F[4,25] = 0.0314, *p* = 0.998; Suppl. Fig. 3).

### Fraction “K” appears to affect 5-HT turnover in brain structures of Sub mice

**Figure 6:**
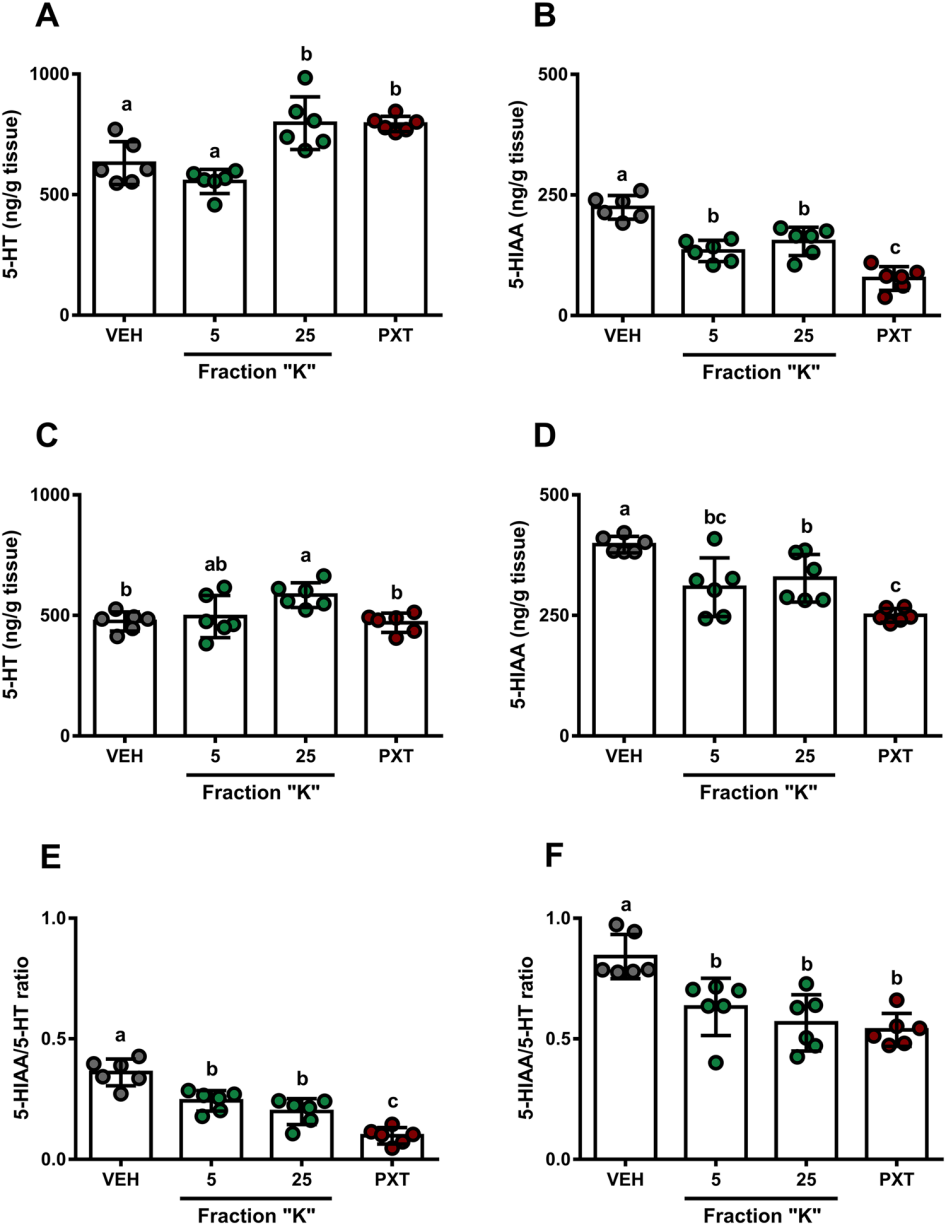
Regional brain tissue levels of 5-HT and 5-HIAA following fraction “K” and PXT treatments. (**A**,**B**,**E**) Prefrontal cortex. (**C**,**D**,**F**) Hippocampus. Mean (± SD; ng/g tissue) levels of prefrontal cortex (**A**) 5-HT, (**B**) 5-HIAA, and (**E**) 5-HIAA/5-HT ratios follow doses of fraction “K” (mg/kg dose indicated, all panels) or PXT (10 mg/kg, all panels). Mean (± SD; ng/g tissue) levels of hippocampus (**C**) 5-HT, (**D**) 5-HIAA, and (**F**) 5-HIAA/5-HT ratios follow doses of fraction “K” (mg/kg dose indicated, all panels) or PXT (10 mg/kg, all panels). One-way ANOVA followed by a Tukey’s HSD: *p* < 0.0001, all panels. Different letters indicate significant differences (*p* < 0.0332).

Neurochemical profiling following a single administration of pharmacologically-active doses of fraction “K” (5 and 25 mg/kg) using HPLC-ECD indicated a dose-dependent decrease in 5-HT content (One-way ANOVA with Tukey’s HSD: F[3,20] = 14.93, *p* < 0.0001, Fig. 6A; F[3,20] = 5.07, *p* = 0.0090, Fig. 6C). Doses of 25 mg/kg fraction “K” produced increases in 5-HT content comparable to PXT in prefrontal cortex (PFC), whereas the same dose produced increased 5-HT content in hippocampus (HPC), yet the dose of PXT used here did not. Concordantly, fraction “K” treatments reduced 5-HIAA content in both PFC (One-way ANOVA with Tukey’s HSD: F[3,20] = 34.62, *p* < 0.0001; Fig. 6B) and HPC (One-way ANOVA with Tukey’s HSD: F[3,20] = 13.22, *p* < 0.0001; Fig. 6D), with PXT treatment showing a more potent effect. Brain region-specific differences in PFC and HPC serotonin content were measured for all treatments, including vehicle control (5-HIAA/5-HT ratios. Two-way ANOVA: Brain region, F[1,40] = 339.2, *p* < 0.0001; treatment, F[3,40] = 28.38, *p* < 0.0001; interaction, F[3,40] = 1.239, *p* = 0.3081; Sidak test: *p* < 0.0001, all treatments; Fig. 6E,F).

### Phytochemical analysis of Fraction “K”

**Figure 7:**
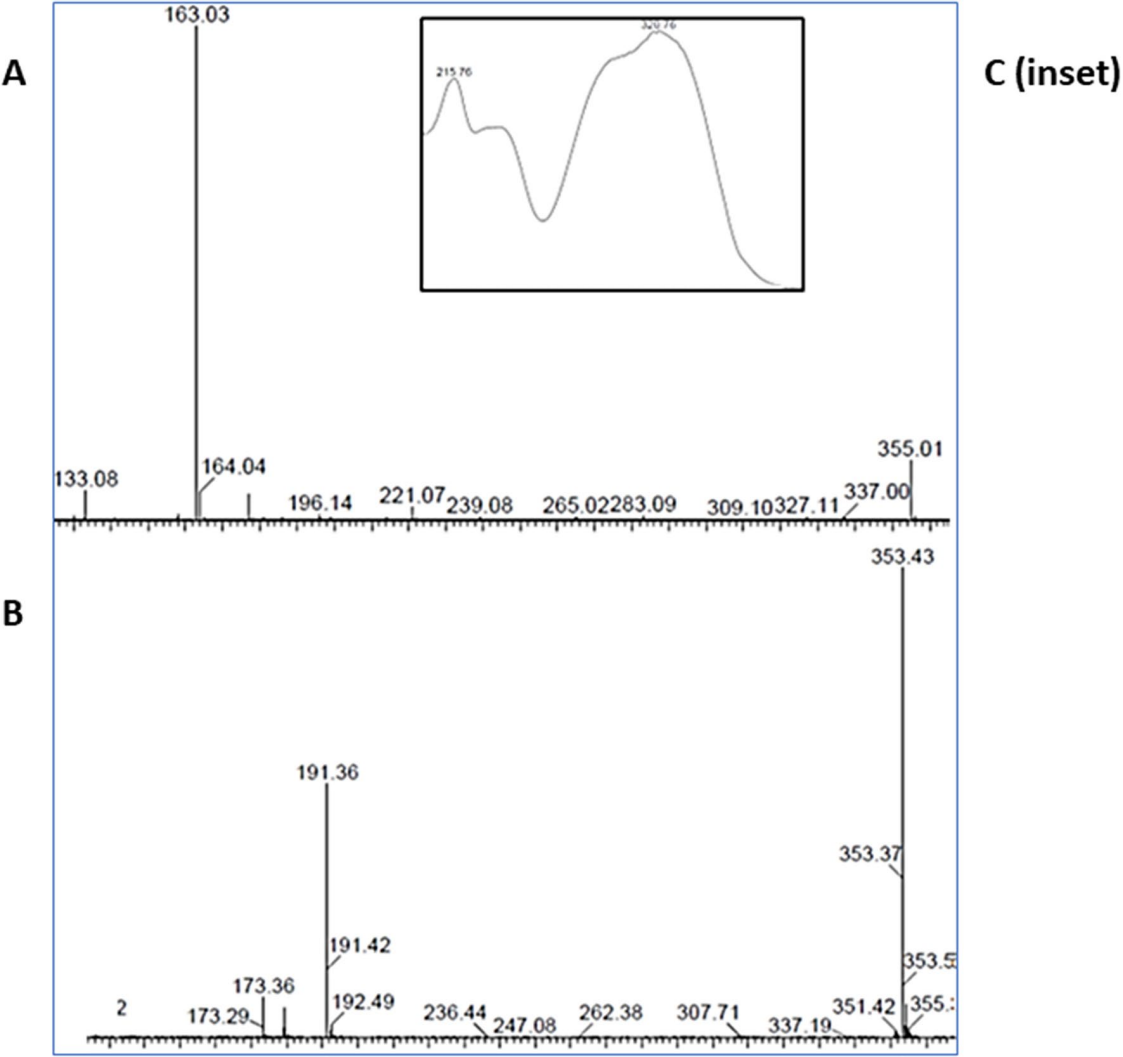
Representative MS and UV spectra of Fraction “K” containing chlorogenic acid and cynarine. (**A**) ESI + mass spectra. (**B**) ESI—mass spectra. (**C**) UV spectra.

Two major components in fraction “K”, representing approximately 87% of the total content, were putatively identified based on positive and negative mass spectra as 49% chlorogenic acid (CGA) and 38% cynarine (Fig. 7A–C).

## Discussion

Among the fractions from *D. viscosa* crude extract, we highlighted one fraction which we entitled “K”, that exhibited a strong cytoprotective effect by dose-dependently inhibiting H_2_O_2_-induced PC12 cell death determined by mitochondrial functionality. Since H_2_O_2_ is a potent apoptosis inducer, we may assume that fraction “K”-mediated cytoprotection may have been achieved by a direct anti-apoptotic effect or indirectly through antioxidative mechanisms similar to the effects shown by other plant extracts^29,30^. Although known antidepressants such as amitriptyline and fluoxetine show protective effects in a PC12 cell model^31^, we found that PXT in a non-cytotoxic concentration range for the drug alone produced increased H_2_O_2_-induced PC12 cell death. This is in agreement with other studies showing the cytotoxic activity of PXT against tumor cells of either murine or human origin^32,33^.

Despite the lack of a clear correlation between the cytoprotective effect of herbal/pharmacological agents in vitro and antidepressant activity in behavioral assays in vivo, several studies have reported such phenomena^34,35^. For example, antidepressant properties of *Hypericum perforatum* have been shown in vivo^36,37^ as well as neuroprotective effects observed in a number of in vitro studies^38,39^. We observed an antidepressant-like effect of fraction “K” in Sub mice possessing a strong depressive-like phenotype, which have been previously shown to be responsive to antidepressants, as well as plant-derived agents^26,40^ In our work, FST was applied as an acute environmental trigger for assessment of stress coping strategy in Sub mice.

The decreased immobility in the FST of Sub mice following fraction “K” administration was linked to a reduction in 5-HT turnover in PFC and HPC similar to PXT. PFC and HPC are considered to be hot-spot brain regions of the circuits involved in depression^41^ and are critically involved in the mechanism of SSRI action^42^. Both PFC and HPC are densely innervated by serotonergic fibers and the majority of 5-HT receptor subtypes are expressed in these regions^43,44^.

Thus, we suggest that fraction “K”-induced changes in the serotonergic system are associated with serotonin transporter (SERT) inhibition, similar to the established mechanism of PXT. PXT functions as a potent SERT inhibitor and reduces 5-HT reuptake, leading to a redistribution of 5-HT in favor of the synaptic cleft and a consequent decrease in presynaptic cytosolic concentrations. These events may trigger compensatory reduction of 5-HT degradation due to the disinhibition of the activity of tryptophan hydroxylase, reflected by decreased 5-HIAA content and increased 5-HT production^45^ as demonstrated here by decreased 5-HIAA/5-HT ratio.

We linked the observed biological activity of fraction “K” to CGA and cynarine, which were identified as major components of the fraction. CGA is a polyphenolic secondary metabolite produced by many plant species, including *D. viscosa*^46^. As a potent antioxidant, CGA shows high capability in modulating oxidative stress in cell- and animal-based models^47,48^. CGA is known to penetrate the blood–brain barrier^49^ exhibiting antidepressant and anxiolytic properties in rodent models^50,51^. CGA affects plasma β-endorphin^52^, serum 5-HT, and dopamine^51^ as well as colonic 5-HT levels^53^. It has also been shown in vitro that CGA stimulates axon and dendrite growth and promotes 5-HT release through augmenting synapsin I expression in rat raphe neurons^49^.

Cynarine has a broad repertoire of biological activities. It is a strong antioxidant compound^54,55^ that also produces effects on smooth muscle, which is thought to contribute to reported antihypertensive activity^56,57^. The antioxidant effects of cyanarine are most potently demonstrated in H_2_O_2_ cytotoxicity assays and is most likely the reason for cytoprotective effects of fraction “K” observed here. Cynarine also has the potential for neurological effects as the chemical has demonstrated anticholinesterase activity with a K_i_ in the low nM range^55^, however any potential psychotropic properties have not been studied.

In this work, we demonstrated, for the first time, the link between changes in the brain serotonergic system and behavioral phenotype following treatment with a *D. viscosa* plant root extract fraction (fraction “K”). Although fraction “K” is CGA and cynarine-enriched and despite the predominance of these two compounds in the isolated fraction, fraction “K” is still a crude preparation and we have not yet identified or have assessed the influences of other chemical agents present. Behavioral effects from fraction “K” treatment resemble those reported for CGA, however we cannot exclude the involvement of other, undetermined agents in the preparation. Further work is mandatory to understand the contribution of each compound in the observed biological activity, the contributions of as-yet unidentified compounds in the remaining 13% of the active fraction “K”, and their possible interactions. We believe the biggest challenge in future efforts will be the assessment of multiple chemical combinations from this bioactive fraction and identification of synergism effects. It is our intent to continue the study of *D. viscosa* root to isolate and characterize compounds with antidepressant action and other positive biological properties.

## Conclusions

A CGA-enriched fraction of *D. viscosa* root extract suppressed the deteriorative effects of H_2_O_2_ on PC12 cells. The study provides the first evidence of a marked in vivo antidepressant-like activity of a CGA-enriched fraction of *D. viscosa* root extract in a mouse model of depressive-like behavior through modulation of the serotonergic system. Further studies are warranted to elucidate the contribution of other compounds within the active fraction to its pharmacological properties.

## Materials and methods

### Plant material collection and root extract preparation

Root samples of *D. viscosa* were collected from wild-growing plants in Ariel, Israel (lat 32.105766; long 35.211673) during June 2018. No national permits were required for plant collection (*D. viscosa* is a common, widespread Mediterranean plant and not on any protected species list). Plant authentication was performed by the National Natural History Collections Herbarium at Hebrew University, Jerusalem; a voucher specimen (HUJ-135000) was deposited (http://nnhc.huji.ac.il/).

To prepare the crude extracts, 100 g of *D. viscosa* roots were gently washed, cut into 1.0 cm sections, boiled in 1.0 L of distilled water (1.0 g/ 10 mL) for 1 h, cooled to room temperature, filtered, and frozen at -80 °C until use.

### Extract fractionation

Fractionation of root crude extract was performed using preparative HPLC (CombiFlash EZ Prep) with Unisol (Agela Technologies) reverse phase C18 column (10 µm; 21.2 × 250 mm; 100 Å). A dual mobile phase gradient (mobile phase A [0.05% formic acid] and mobile phase B [acetonitrile]) was used to achieve appropriate separation. The gradient composition B:A was as follows: 5:95% (0 min); 15:85% (0–5 min); 20:80% (5–10 min); 25:75% (10–15 min); 30:70% (15–25 min); 35:65% (25–70 min). The flow rate was 21.0 mL·min^−1^ and the injection volume was 10 mL of crude extract.

Extract was separated into 14 fractions, each combining 5 min of flow, and assigned a letter (A-N). A UV detector visualized the peaks at wavelengths of 280 and 257 nm. Each fraction was lyophilized at − 80 °C for approximately 72 h and reconstituted to an aqueous solution. All reconstituted fractions were standardized to fit the amount of the major parallel peaks in the crude extract by diluting in *n* times (for example, reconstituted fraction “K” was diluted seven times to achieve its initial concentration in crude extract).

Data were collected and analyzed using ChromNAV 2.0 HPLC Software.

### Chemicals and treatments

For in vivo and ex vivo studies, 5 mg/kg *D. viscosa* root crude extract or 1, 5, or 25 mg/kg of isolated fraction were used. The doses were chosen based on our pilot studies that identified pharmacological activity of the crude root extract or isolated fraction within this dose range. Saline was used as a solvent vehicle (VEH). Paroxetine (PXT, Sigma-Aldrich cat. PHR 1804S; 10 mg/kg i.p.), an antidepressant agent, was used for comparison with *D. viscosa* preparations since Sub mice with depressive-like behavior have shown antidepressant effects when treated with this drug^26^. All injections were performed i.p at a volume of 5 μL/g body weight, 60 min before the behavioral or neurochemical studies. For in vitro studies, cells were incubated in the presence of crude extract (250–5000 μg/mL), isolated fractions (250–1000 µg/mL), or PXT (10–100 µM) diluted in RPMI medium.

### H_2_O_2_ cytotoxicity assay

Rat pheochromocytoma cells (PC12; ATCC, CRL-1721 were grown in RPMI medium supplemented with 10% heat-inactivated horse serum, 5% fetal bovine serum, and 0.1% penicillin/streptomycin at 37 °C in a humidified incubator with 5% CO_2_. For all experiments, cells were seeded into 96-well plates at a density of 5 × 10^4^ cells/well/100 µL. Cell protection was assessed using the hydrogen peroxide (H_2_O_2_)-based oxidative stress cytotoxicity model. PC12 cells were treated with 400 µM H_2_O_2_ (IC_50_) for 12 h following 2 h of pretreatment with differing concentrations of *D. viscosa* extracts. Cell viability was determined by the XTT reduction assay (Biological Industries, Cat. No 20-300-1000). The 400 µM concentration was selected to produce 50% cell death as measured in the XTT assay.

### Animals

Submissive (Sub) mice used in this study were selectively bred for 36 generations from an outbred Sabra strain (Envigo laboratories, Israel) using social interaction dominant-submissive relationship paradigm^58^. The behavioral profile of Sub mice possesses strong elements of depressive-like behavior and impaired stress coping strategies confirmed by different experimental approaches^25–28^.

Animals were housed in groups of five in a temperature (21 ± 2 °C), humidity (55 ± 5%), and light controlled room (lights on from 7 AM to 7 PM). Standard laboratory chow and water were available ad libitum. All experiments were conducted with male mice (age, 10 weeks), during the light phase of the day-night cycle between 9 AM and 3 PM. Behavioral and neurochemical studies were conducted on the separate cohorts of Sub mice. Housing, care, and experimental procedures involving animal use conformed to NIH/USDA and ARRIVE 2.0 guidelines. The experiments were approved and supervised by the Institutional Animal Care and Use Committee of Ariel University and the Israel Ministry of Health (Protocol IL-74-09-15).

### Open field test (OF)

Spontaneous locomotor (horizontal) activity was assessed in the Open Field (OF) test using EthoVision (Noldus, Holland) as previously described^27,59^. Briefly, the OF apparatus consisted of a square black plastic chamber (40 cm × 40 cm). Each mouse was placed individually for 5 min in the center of the chamber and distance travelled was recorded. Between subjects, the apparatus was thoroughly washed with 70% ethanol and dried with tissue paper.

### Forced swim test (FST)

FST was performed to access stress coping strategies following psychotropic interventions^60^. Mice (6 per group) were individually tested in a glass cylinder (30 cm in height, 10 cm in diameter) filled with water up to 25 cm height (25 ± 2 °C). Mice were tested for 6 min, the last 4 min were analyzed, where the total time spent immobile was recorded manually. Mice were considered “immobile” if they displayed no activity except for what is required to keep the head above water.

### Neurochemical profiling

For sample preparation, mice were anesthetized in a CO_2_ chamber and decapitated immediately afterwards. Prefrontal cortex (PFC) and hippocampus (HPC) were dissected, frozen in liquid nitrogen and kept at − 80 °C until use. Serotonin (5-hydroxytryptamine, 5-HT) and its metabolite 5-hydroxyindoleacetic acid (5-HIAA) were assayed in brain tissue samples using HPLC with electrochemical detection (EICOM Co., Kyoto, Japan) as previously described^45^.

### HPLC–MS/MS analysis

Chemical analysis of fractions was performed using a Waters 2695 Separation Module with a Photodiode Array Detector together with a Quattro Micro Mass Spectrometer. Chromatographic separation was achieved using a Luna (Phenomenex) reverse phase C18 (5 µm; 4.6 × 250 mm; 100 Å) column and the binary gradient described above (see Extract fractionation). MS acquisition was conducted in both ESI positive and negative ionization mode under the following conditions: capillary voltage—3.5 kV, cone voltage—45 V, extractor voltage − 3 V, RF lens − 0.2 V, source temperature − 120 °C, desolvation temperature − 350 °C, nitrogen flow rate of 700 L/h for desolvation and 50 L/h cone gas. Tentative identification of compounds was achieved based on comparisons with purchased standards (Cayman Chemical Company) as wells as spectra from an MS library (NIST 2017).

### Statistical analysis

Data are expressed as means (± SD). All multiple comparisons were Bonferroni-corrected against α = 0.05 and all analyses were two-tailed. Multiple comparison analyses were performed by one-way ANOVA followed by a Tukey’s HSD or Dunnett’s test as appropriate or were compared by two-way ANOVA followed by a Sidak or Tukey’s HSD test based on the structure of the dataset. All analyses were performed in GraphPad Prism 7.0. Results of all analyses are presented in detail in Supplementary Table S1. Data from this study are publicly available in the Mendeley Data repository^61^.

## Supplementary Information

Supplementary Figures.

Supplementary Table S1.
