# Supplementary material for: Antidepressant-like effects of a chlorogenic acid- and cynarine-enriched fraction from Dittrichia viscosa root extract
Source: Sci Rep. 2022 Mar 7;12:3647. doi: 10.1038/s41598-022-04840-9 (PMC8901669; doi:10.1038/s41598-022-04840-9)
Supplement: Supplementary file 1 — Supplementary Figures. [file 41598_2022_4840_MOESM1_ESM.pdf]

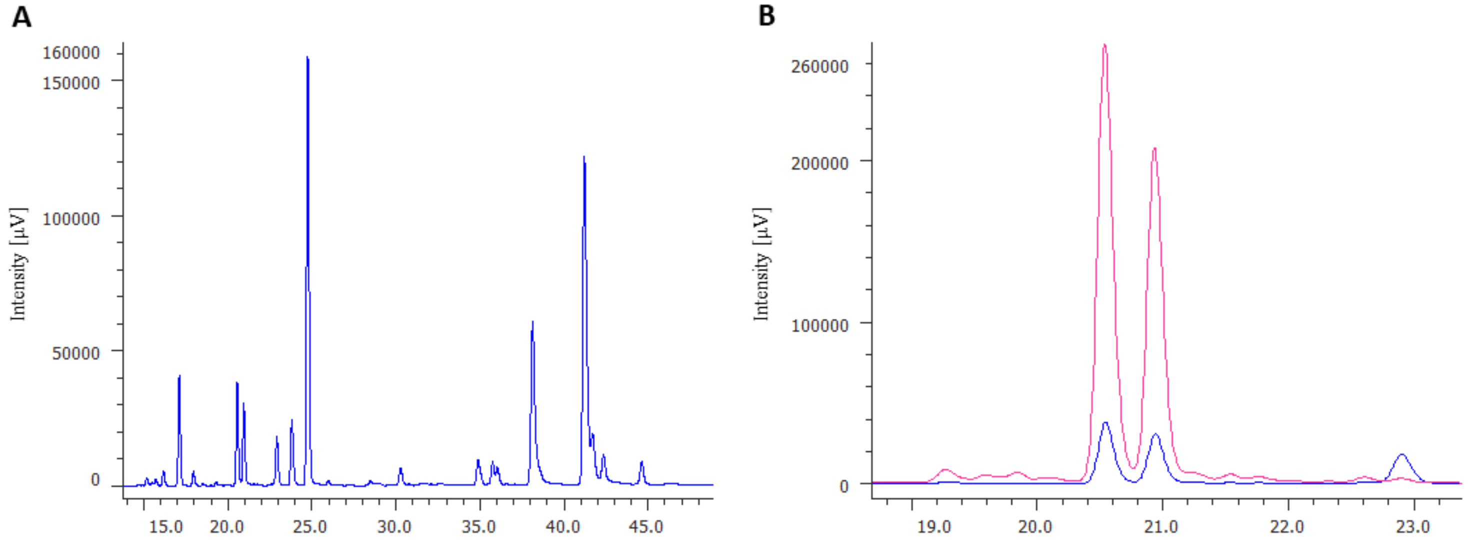

Suppl. Fig. 1. HPLC chromatograms of crude root extract (A) and fraction "K" (B) used for *in vitro* and *in vivo* studies, visualized at 280 nm. Chromatogram peaks of the crude extract (**A, B**; blue). Chromatogram peaks of the fractionation product entitled "K" (**B**; red).

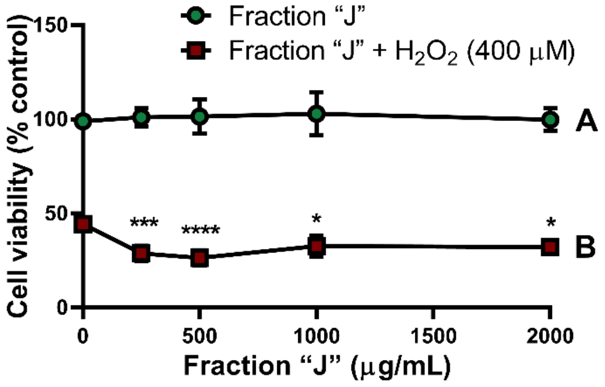

Suppl. Fig. 2. Cytotoxic effect of fraction "J" on PC12 cells under H_2O_2 cytotoxicity. Mean cell viability (±SD) measured by XTT assay for (**A**) PC12 cells incubated with concentrations of fraction "J" (0-2000 µg/mL) for 14 h or (**B**) PC12 cells pretreated with concentrations of fraction "J" (0-2000 µg/mL) for 2 h followed by incubation for 12 h with H_2O_2 (400 µM). Two-way ANOVA: concentration, $p=0.0264$; H_2O_2 treatment, $p<0.0001$; interaction, $p=0.0016$. Dunnett's test: *, $p<0.016$, ***, $p=0.0005$, ****, $p<0.0001$.

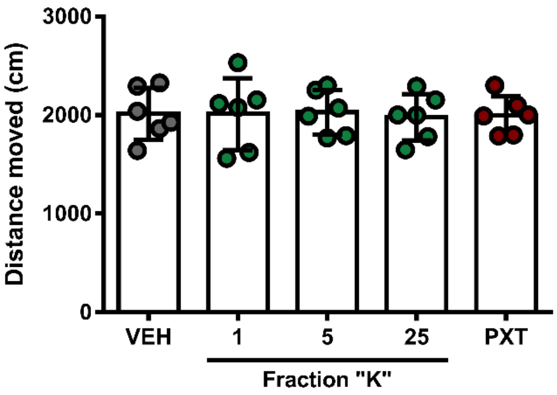

Suppl. Fig. 3. Locomotory activity with fraction "K" treatment in the OF test. Mean distance moved (±SD, cm) in 5 min following treatment with doses of fraction "K" (mg/kg) or PXT (10 mg/kg). One-way ANOVA, $p>0.05$.
